# Two–three parameters isotherm modeling, kinetics with statistical validity, desorption and thermodynamic studies of adsorption of Cu(II) ions onto zerovalent iron nanoparticles

**DOI:** 10.1038/s41598-021-95090-8

**Published:** 2021-08-12

**Authors:** Adewumi O. Dada, Folahan A. Adekola, Ezekiel O. Odebunmi, Adeniyi S. Ogunlaja, Olugbenga S. Bello

**Affiliations:** 1 Nanotechnology Laboratory, Industrial Chemistry Programme, Department of Physical Sciences, Landmark University, P.M.B.1001, Omu-Aran, Kwara State Nigeria; 2 Department of Industrial Chemistry, University of Ilorin, P.M.B. 1515, Ilorin, Nigeria; 3 Department of Chemistry, University of Ilorin, P.M.B. 1515, Ilorin, Nigeria; 4 Department of Chemistry, Nelson Mandela Metropolitan University, P.O. Box 77000, Port Elizabeth, 6031 South Africa; 5 Department of Pure and Applied Chemistry, Ladoke Akintola University of Technology, P.M.B 4000, Ogbomosho, Oyo State Nigeria; 6 Landmark University, Sustainable Development Goal (SDG) Group 6: Clean Water and Sanitation, Omu-Aran, Nigeria; 7 Landmark University, Sustainable Development Goal (SDG) Group 11: Sustainable Cities and Communities, Omu-Aran, Nigeria

**Keywords:** Materials science, Nanoscience and technology

## Abstract

Adsorption of problematic copper ions as one of the endocrine disruptive substances from aqueous solution onto nanoscale zerovalent iron (nZVI) was studied. The high pore size 186.9268 Å, pore diameter 240.753 Å, and BET surface area 20.8643 m^2^ g^−1^ and pH(pzc) enlisted nZVI as an efficient nano-adsorbent for treatment of heavy metals from synthetic wastewater. SEM and EDX revealed the morphology and elemental distribution before and after adsorption. 98.31% removal efficiency was achieved at optimum adsorption operational parameters. Of all the thirteen isotherm models, equilibrium data were well fitted to Langmuir. Kinetics and mechanism data across the concentrations from 10 to 200 mg L^−1^ were analyzed by ten models. PSO best described kinetics data as confirmed by various statistical error validity models. The intraparticle diffusion model described that the intraparticle diffusion was not the only rate-limiting step. The adsorption mechanism was diffusion governed established by Bangham and Boyd models. Feasible, spontaneous, endothermic, and degree of randomness were reveal by the thermodynamic studies. Better desorption index and efficiency were obtained using HCl suggesting multiple mechanism processes. The performance of ZVI suggested it has a great potential for effective removal of endocrine disruptive cationic contaminant from wastewater.

## Introduction

Endocrine disruptive compounds (EDC) have been among emerging contaminants whose adverse effects in the environments have received the attention of various researchers. Heavy metal ions have been identified to have hazardous effects on the endocrine system. Copper, as heavy metal ions, has been listed among endocrine disruptive compounds. Some of the endocrine disruptive effects of copper ions are: Increased levels of plasma cortisol associated with protein synthesis, cell proliferation, and apoptose in gill cells. It can also lead to an increase in Catecholamines which promotes metabolic and haematopoietic responses^1,2^. Several anthropogenic activities and natural phenomena release copper ions into the environment^3^. The largest threat to human lives and aquatic organisms arises from the soluble form of copper^4^. These soluble copper ions get released into the environment via different agricultural applications. Research has shown that high uptakes of copper may cause liver and kidney damage and even death^5–7^. Research on the utilization of nanoparticles is on the increase due to their special characteristics. Nanotechnology is a trending research area for the science and technology of functional structures at the molecular scale. This covers current research work in chemical, physical, biological, medical, material sciences, and engineering. Nano-materials have been reported to be applicable in environmental remediation, catalysis, development of optical devices, and medicine^8^. Nanoparticles are the new trend of effective adsorbents used in the decontamination of water and immobilization of heavy metal ions from their solutions^9^. A study conducted by the U.S Environmental Protection Agency showed that zerovalent iron nanoparticle (nZVI) is environmentally benign and effective in soil and water remediation^8,10–12^. nZVI has found relevance in the adsorption of problematic toxicants such as EDC heavy metal ions. Of all various conventional approaches^13^ described for heavy metal ions removal, adsorption via adsorption is much more favored since it is low cost, efficient, available, and easy to operation^14–16^.

The most vital quantity for comprehending the adsorption process is gotten from adsorption isotherm models. The parameters of isotherm modeling are essential factors relevant to the design of an effluent treatment reactor. More so, extensive isotherm models were investigated to predict and compare adsorption performances. Most common isotherm models are Langmuir and Freundlich, Temkin and Dubinin–Kaganer–Raduskevich (DKR). Other isotherm models used in this study are Halsey, Jovanovic, Elovich, Jossen, Flory–Huggins, Kiselev, Harkins–Jura, Fowler–Guggenheim, and Redlich–Peterson. In most adsorption studies carried out using nZVI, no detailed investigation has been reported for mathematical isotherm parameters that could be utilized for treatment reactor design. This has not been given a priority hence a research gap of global interest has been created. The energy of the adsorption process for uptake of endocrine disruptive copper ions via thermodynamic studies was examined to determine the feasibility, spontaneity, energy content, and degree of disorderliness of the process. More so, the mechanism vis-à-vis desorption studies were investigated using three desorbing agents. The reality of the adsorption process was assessed by post-adsorption characterization using Fourier Transform Infrared Spectroscopy (FTIR), Scanning Electron Microscopy (SEM), and Energy Dispersive X-ray (EDX).

## Materials and methods

All through this work, analytical grade reagents were used without further purification. Double-Distilled-Deionized water, Copper sulphate (CuSO_4_·5H_2_O, Breckland Scientific Batch No. 6688), Isopropyl alcohol (BDH, Min. Assay 99%, Prd No. 29694 6). Other chemicals purchased from Sigma Aldrich, USA are Sodium borohydride (NaBH_4_), Iron (III) chloride (FeCl_3_.6H_2_O), Hydrochloric acid (HCl), Sodium hydroxide (NaOH), Sodium nitrate (NaNO_3_).

### Synthesis of zerovalent iron nanoparticles (nZVI)

The synthesis of nZVI for the removal of endocrine disruptive heavy metal ions was undertaken by following the procedure described in our prior studies^6,17^. Under an anaerobic environment, a resulting black coloration of core–shell zerovalent iron nanoparticles (nZVI) was obtained from the reaction between 0.023 M solution of FeCl_3_·6H_2_O and 0.125 M solution of NaBH_4_ in ratio 1:5. Detailed synthetic procedure is presented in the supplementary document associated with this study. The synthesis equation is depicted in Eq. (1):

1 $$4{\text{Fe}}^{3 + } + 3{\text{BH}}_{4}^{ - } + 9{\text{H}}_{2} {\text{O}} \to 4{\text{Fe}}^{0} \downarrow + 3{\text{H}}_{2} {\text{BO}}_{3}^{ - } + 12{\text{H}}^{ + } + 6{\text{H}}_{2} \uparrow$$

### Surface charge (pH_pzc_), BET surface area, surface morphology and elemental distribution

Following the salt addition and pH variation method, the point of zero charge was determined as presented in the supplementary materials^18^. Surface area by BET, pore width, and volume were determined using Micrometritics AutoChem II Chemisorption Analyzer. The surface morphological characterization and elemental analysis were carried out using a Scanning Electron Microscopy (SEM) integrated with Energy Dispersive X-ray (EDX) analyzer. SEM images and EDX spectra were obtained using a TESCAN Vega TS 5136LM typically at 20 kV at a working distance of 20 mm. Samples for SEM analysis were prepared by coating them in gold using a Balzers’ Spluttering device.

### Effect of stirring speed, pH, and co-existing ions

In order to optimize the stirring speed, 160–240 rpm speed was studied at optimum conditions. Effect of pH was studied by regulating the solution to the desired pH value using 0.1 M NaOH and 0.1 M HNO_3_ solutions. Effect of Co-existing ions/Ionic strength varying the concentration of NaCl introduced into Cu^2+^ solution from 0.001 to 1.0 M.

### Batch isotherm, kinetics, and thermodynamic studies

A typical batch adsorption study was carried out following procedure reported in our previous study^19,20^. 1000 ppm Cu^2+^ stock solution was prepared by dissolving 2.5 g of CuSO_4_.5H_2_O in 1000 mL of distilled-deionized water. Study on initial Cu^2+^ ion concentration was examined by adding 100 mg nZVI at different Cu (II) ions concentrations (10–200 ppm) and residual concentration determined by using AAS model AA320N. The quantity adsorbed and percentage removal efficiency were calculated utilizing Eqs. (2) and (3)^21–23^:

2 $$Q = \frac{{(c_{o} - c_{e} )V}}{m}$$

3 $$\% \;RE = \frac{{C_{i} - C_{e} }}{{C_{i} }} \times 100$$

The characteristics and mechanism of the adsorption process were investigated from the study of the Ce-dependent changes of Qe applied to thirteen isotherm models. Similarly, the batch adsorption kinetic experiments were conducted at optimum conditions for contact time ranging from 10 to 120 min. Adsorption capacities at contact time were obtained using Eq. (4)^5,23^:

4 $$Q_{t} = \frac{{(C_{o} - C_{e} )V}}{W}$$

Kinetic data were fitted to ten kinetics and mechanism models.

From the thermodynamics studies, the effect of temperature at optimum conditions was investigated at five different temperatures (298 K, 303 K, 318 K, 328 K, 333 K) for adsorption of endocrine disruptive Cu^2+^ onto nZVI following our previously reported procedure^24,25^. The study was carried out in a temperature-controlled water bath. Data obtained were fitted to Van’t Hoff equation depicted in Eqs. (5) and (6).

5 $$logK_{C} = \frac{\varDelta S^\circ }{{2.303R}} - \frac{\varDelta H^\circ }{{2.303RT}}$$

6 $$\varDelta G = - 2.303RT\ell ogKc$$

R is the gas constant (8.314 J mol^−1^ K)^−1^), T the absolute temperature (K), K_c_ (q_e_/Ce) an equilibrium constant at various temperature. Standard enthalpy change ∆H° (kJ mol^−1^) and standard entropy change ∆S° (J mol^−1^ K^−^1) were determined from the slope and intercept of the Van’t Hoff plot of log K_c_ versus 1/T^26,27^.

### Desorption studies

The desorption is a means of regenerating the adsorbent capacity for reusability and cost effectiveness determination. Desorption studies were investigated using the following eluents: deionized (DI) water, 0.2 M HCl and 0.2 M CH_3_COOH, at pre-determined optimum conditions. The desorption capacity, percentage desorbed, desorption efficiency and desorption index were determined using Eqs. (7)–(10)^28^:

7 $$q_{des} = C_{des} \frac{V}{W}$$

8 $$\% \;Desorption\; = \;\frac{{C_{des} }}{{C_{i} }} \times 100$$

9 $$\% \;Desorption\;Efficiency\; = \;\frac{{q_{des} }}{{q_{e} }} \times 100$$

10 $$Desorption\;index\; = \;\frac{\% \;total\;metal\;removed\;after\;adsorption}{{\% \;total\;metal\;remained\;on\;the\;adsorbent\;after\;desorption}}$$

where q_des_ is the quantity of metal ion desorbed (mg g^−1^), q_e_ is the quantity of metal adsorbed after sorption (mg g^−1^), C_des_ is the concentration of metal ion left after desorption (mg L^−1^), V is the volume of the metal ion solution (mL) while W is the weight of adsorbents (mg).

## Results and discussion

### nZVI physicochemical characterization: pH(pzc), BET surface area, pore volume and pore size

Summarized in Table 1 are pH, point of zero charge (PZC), BET surface area, and other physicochemical parameters describing the core–shell. The point of zero charge finds relevance in surface and nanoscience.

**Table 1:** Physicochemical parameters of nZVI.

Physicochemical parameters	nZVI
pH	6.80
PZC	5.84
BET surface area	20.8643 m^2^ g^−1^
t-Plot micropore area	4.4140 m^2^ g^−1^
t-plot external surface area	16.4503 m^2^ g^−1^
BJH adsorption cumulative surface area of pores	19.120 m^2^ g^−1^
**Pore volume**	
Single point adsorption total pore volume of pores	
less than 1103.482 Å diameter at P/Po = 0.982136052:	0.097502 cm^3^ g^−1^
t-Plot micropore volume:	0.001895 cm^3^ g^−1^
BJH adsorption cumulative volume of pores	0.115083 cm^3^ g^−1^
**Pore size**	
Adsorption average pore width (4 V/A by BET):	186.9268 Å
BJH Adsorption average pore diameter (4 V/A):	240.753 Å

Figure S1 (from the supplementary document associated with this study) shows the pH(pzc) of nZVI. It revealed that adsorption of Cu^2+^ would take place at a pH > pH_pzc_ as a result of more active binding being available due to deprotonation and low electrostatic repulsion. This finding shows that nZVI was positive at pH < pH_pzc_ and negative at pH > pH_pzc_. Thus this signposts the suitability of nZVI for effective adsorption. The BET surface area 20.86 m^2^ g^−1^ and the external surface area 16.4503 m^2^ g^−1^ being greater than their corresponding micropores further support the suitability of nZVI for adsorption. Higher surface area enhances the adsorption process as supported. Therefore, it can be deduced that nZVI nano-adsorbent would utilize its external surfaces for heavy metal uptake than its micropore areas^29^.

### SEM/EDX characterization

**Figure 1:**
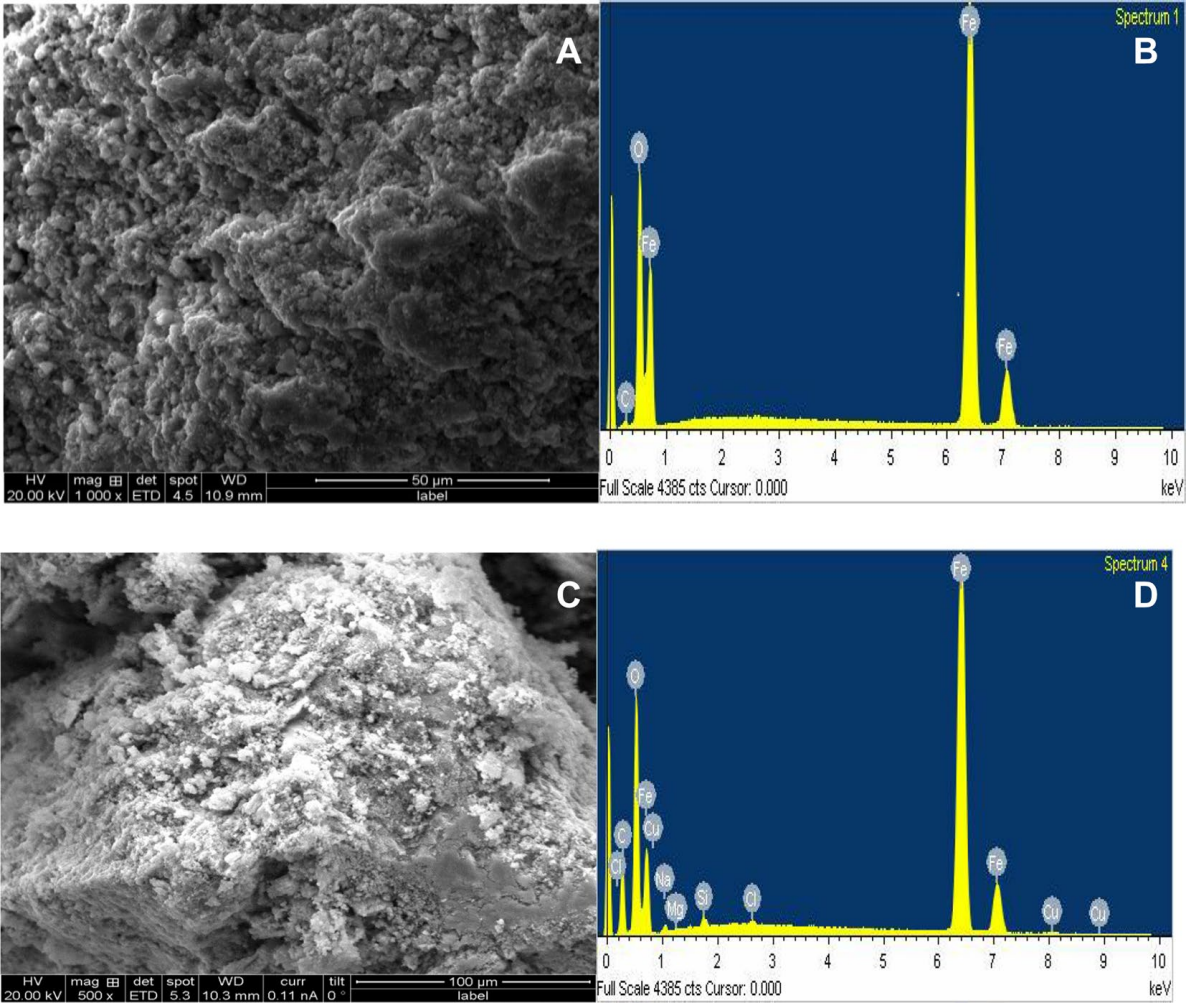
(**A**) SEM micrograph of nZVI before adsorption. (**B**) EDX spectrum of nZVI before adsorption. (**C**) SEM micrograph of Cu-loaded-nZVI after adsorption. (**D**) EDX of Cu-loaded-nZVI after adsorption.

Percolation of EDC-Cu^2+^ into the pores ad matrix of nZVI was proved by the SEM/EDX depicted in Fig. 1A–D. Figure 1A revealed the SEM image before adsorption while Fig. 1B depicted the EDX with an intense peak of a zerovalent iron nanoparticle. Before adsorption spherical, chain-like aggregated morphology of nZVI was revealed SEM. The core–shell nature of zerovalent iron with intense peaks between 0.6–6.4 and 7.0 keV was revealed from the EDX result in Fig. 1B. Presented in Fig. 1C is the SEM micrograph showing swollen and robust nature of the surface of the nZVI nano-adsorbent after adsorption suggesting that the nZVI surface had been Cu-loaded up. More so, corroborating the result from SEM, Fig. 1D revealed the EDX spectrum showing the presence of Cu(II) as evidence of Cu adsorption onto core–shell nZVI. This is supported by finding in the literature^30^.

### Effect of operational parameters

#### Effect of initial Cu^2+^ concentration

A major role in the adsorption study is played by the factor of initial concentration at optimum conditions presented in supplementary document as seen in Figure S2 This showed that at optimum conditions, 85.04% RE and 81.04 mgg^−1^ quantity of Cu^2+^ was adsorbed. The extent of removal of Cu(II) ion cation increased based on the availability of more active sites at lower concentrations until the pore sizes were saturated at an advanced concentration (150–200 mg L^−1^). Concentration gradient was built up in the Cu-nZVI system due to intensification of drive force as concentration increased from 10 to 200 mg L^−1^. This is supported by the findings in the literature^31^.

#### Effect of contact time

The Build-up of Cu(II) ions at the solid–liquid interfaces are controlled by the contact time. From this study, optimization of the contact time was investigated from 10 to 120 min. Fast kinetics from the bulk to the outer and inner surface of the nano-material (nZVI) identified by a short contact time to reach equilibrium was observed in the supplementary document as seen in Figure S3 Quantity of Cu(II) adsorbed increase from 4.96 to 82.82 mg g^−1^ as the initial Cu^2+^ concentration increased from 10 to 200 ppm. A similar trend was observed by Baby et al.^32^ on the adsorption of heavy metals.

#### Effect of initial solution pH

The key to the adsorption of heavy metal ions is the solution pH because it affects the surface chemistry of the system. A plot of the effect of initial concentration is presented in the supplementary document as seen in Figure S4 portrayed the effect of pH at optimum conditions. Coined from the understanding of the isoelectric point of the pH(pzc), nZVI is suitable for the uptake of cationic pollutants such as Cu^2+^ since the pH > pH(pzc). At low solution pH, the system is protonated leading to electrostatic competition among Cu^2+^ and other cationic species such as H^+^, Cu(OH)^+^, Cu(OH)_2_. However, at solution pH > pH(pzc), the system is negative, deprotonation occurs, there is less competition between Cu^2+^ and other anionic species (Cu(OH)_3_^−^ and Cu(OH)_4_^2−^). Effective adsorption occurs at pH > pH(pzc). Optimum adsorption was achieved at pH 6 with 98.31% removal efficiency and quantity adsorbed 73.73 mg g^−1^ indicating effective binding of Cu^2+^ onto nZVI surface. This is corroborated by the findings of other researchers^33^.

#### Effect of ionic strength

Analysis of Figure S5 (presented in the supplementary document associated with this study) showed the effect of ionic strength on Cu^2+^ adsorption. Pollution of the water system is not limited to heavy metal ions only, some co-existing ions increase the salinity and ionic strength of the water body as investigated in this study. Co-existing ions polluted waste system increases the salinity and background electrolyte of the water body. A decrease in the percentage of Cu^2+^ removed from 81.99 to 79.73% with a reduction in quantity adsorbed from 61.49 to 59.79 mg g^−1^ was observed in Figure S5. The decrease in Cu(II) ions uptake may also be due to a decrease in the electrostatic attraction arising from compressed electrical diffuse double layer supporting the findings of Advantageously, the removal efficiency of 81.99% shows that nZVI is an effective nano-sorbent in treatment industrial discharge containing co-existing ions. This is supported by findings in the literature^34^.

#### Effect of stirring speed

This study demonstrated as shown in Figure S6 (supplementary document) that at 200 rpm maximum adsorption of Cu^2+^ onto nZVI was attained. Stirring speed is also one of the important parameters in adsorption studies because it promotes turbulence, frequency of collision and improves mass transfer in the medium between the two phases. At 200 rpm, the percentage Cu^2+^ removal efficiency and quantities adsorbed are 96.98% and 72.73 mg g^−1^ for nZVI. Stirring speed increases the retention of Cu^2+^ and it encourages a better transfer of Cu^2+^ between solid–liquid interfaces (Cu^2+^-nZVI system)^18^. No appreciable percentage removal efficiency was observed after 200 rpm and all other study was carried out at this stirring speed.

### Two–three parameters adsorption isotherm modelings

One of the important and significant aspects of adsorption studies is mathematical isotherm modeling. Isotherm modeling is important in order to observe the relationship between nZVI and Endocrine disruptive Cu(II) ions at equilibrium conditions. A good understanding of this would significantly enhance the design of the adsorption system, effluent treatment reactor, and the pattern describing adsorbate-adsorbent interaction. Equilibrium data obtained from initial concentration were analyzed using thirteen mathematical isotherm models. All mathematical isotherm models used in this study were presented in Table 2 together with their non-linear, linear equations and parameters' description. The estimated parameters are portrayed in Table 3. The plots in the isotherm studies are presented in the supplementary document associated with this article from Figure S7A–S7M.

**Table 2:** Adsorption isotherm and kinetics models^35,43,47^.

Types of adsorption models	Non-linear expression	Linear expression	Parameters nomenclature and description
Langmuir	$Q_{e} = \frac{{Q_{max} K_{L} C_{e} }}{{1 + K_{L} C_{e} }}$	$$\begin{array}{*{20}l} {\frac{{{\varvec{Ce}}}}{{{\varvec{Q}}_{{\varvec{e}}} }} = \frac{{{\varvec{Ce}}}}{{{\varvec{Q}}_{{{\varvec{max}}}} }} + \frac{1}{{{\varvec{Q}}_{{{\varvec{max}}}} {\varvec{K}}_{{\varvec{L}}} }}} \hfill & {\left( {11} \right)} \hfill \\ {{\text{R}}_{{\text{L}}} = \frac{1}{{1 + K_{L} C_{o} }}} \hfill & {\left( {12} \right)} \hfill \\ \end{array}$$	K_L_ is the Langmuir isotherm constant (L mg^−1^) related to the binding energy of adsorption.$Q_{max}$ is the maximum monolayer coverage capacity (mg g^−1^) R_L_ dimensionless separation factor indicating the nature and favourability of adsorption process. From slope and intercept of linear plot of Ce/Qe versus Ce, K_L_ and Q_max_ were determined
Freundlich	$Q_{e } = K_{F} C_{e}$	$$\begin{array}{*{20}l} {logQ_{e} = logK_{F} + \frac{1}{{n_{F} }} logC_{e} } \hfill & {\left( {13} \right)} \hfill \\ \end{array}$$	*C*_*e*_ equilibrium concentration of the MG dye adsorbate (mg L^−1^); Q_e_ amount of MG dye adsorbed at equilibrium per unit weight of nZVI (mg g^−1^); *K*_F_ Freundlich indicator of adsorption capacity 1/n_F_ Intensity of the adsorption indicating the surface heterogeneity and favourability of the adsorption process. 1/n_F_ and *K*_F_ were determined from slope and intercept of linear plot of log Qe versus log Ce
Temkin	$Q_{e} = \frac{RT}{{b_{T} }} ln\left( {A_{T} C_{e} } \right)$	$$\begin{array}{*{20}l} {Q_{e} = \frac{RT}{{b_{T} }}lnA_{T} + \frac{RT}{{b_{T} }}lnC_{e} } \hfill & {\left( {14} \right)} \hfill \\ \end{array}$$	***b***_***T***_ is the Temkin isotherm constant related to the heat of adsorption and A_*T*_ is the Temkin isotherm equilibrium binding constant (L g^−1^) R = universal gas constant (8.314 J mol^−1^ K^−1^) T = absolute Temperature in Kelvin B = RT/b_T_ = constant related to heat of sorption (J mol^−1^) obtained either from intercept or slope
DKR	$Q_{e} = Q_{DKR} exp^{{ - A_{D - R} }} \varepsilon^{2}$	$$\begin{array}{*{20}l} {\ell nq_{e} = \ell nQ_{DKR} - A_{DKR} \varepsilon^{2} } \hfill & {\left( {15} \right)} \hfill \\ {\varepsilon = RT\ell n\left[ {1 + \frac{1}{{C_{e} }}} \right]} \hfill & {\left( {16} \right)} \hfill \\ {E = - \left[ {\frac{1}{{\sqrt {2A_{D - R} } }}} \right]} \hfill & {\left( {17} \right)} \hfill \\ \end{array}$$	Q_DKR_ is the theoretical adsorption isotherm saturation capacity (mg g^−1^) obtained from intercept. A_DkR_ is the D–R isotherm constant (mol^2^ kJ^−2^) related to free sorption energy obtained from the slope. Ɛ is Polanyi potential determined by the expression = RT ln(1 + 1/C_e_). E is the mean adsorption free energy helpful in determining the adsorption nature (physisorption or chemisorption of the adsorption process). Q_D–R_ and A_D–R_ were determined from intercept and slope of linear plot of ln q_e_ versus Ɛ^2^
Halsey	${\varvec{Q}}_{{\varvec{e}}} = {\varvec{exp}}\left[ {\frac{{{\varvec{lnK}}_{{\varvec{H}}} - {\varvec{lnC}}_{{\varvec{e}}} }}{{{\varvec{n}}_{{\varvec{H}}} }}} \right]\user2{ }$	$$\begin{array}{*{20}l} {log\;Q_{e} = \left[ {\left( {\frac{1}{{n_{H} }}} \right) lnK_{H} } \right] - \left( {\frac{1}{{n_{H} }}} \right) lnC_{e} } \hfill & {\left( {18} \right)} \hfill \\ \end{array}$$	K_H_ is Halsey isotherm constant; n_H_ is the Halsey isotherm exponent. Both were determined from linear plot of logQe versus ln Ce
Harkin–Jura	$Q_{e} = \left( {\frac{{A_{HJ} }}{{B_{HJ} - logC_{e} }}} \right)^{\frac{1}{2}}$	$$\begin{array}{*{20}l} {\frac{1}{{Q_{e}^{2} }} = \frac{{B_{HJ} }}{{A_{HJ} }} - \frac{1}{{A_{HJ} }}\ell ogCe} \hfill & {\left( {19} \right)} \hfill \\ \end{array}$$	A_H_ is the Harkins–Jura isotherm parameter B_2_ is the Harkins–Jura isotherm constant The Harkin–Jura constants, A_HJ_ and B_HJ_, were determined from the slope and intercept of the linear plot of 1/Qe^2^ versus logCe
Redlich–Peterson	$Q_{e} = \frac{{A_{R - P} Ce}}{{1 + BC_{e}^{\beta } }}$	$$\begin{array}{*{20}l} {\ln \left( {\frac{{C_{e} }}{{Q_{e} }}} \right) = \beta \ln C_{e} - \ln A_{R - P} } \hfill & {\left( {20} \right)} \hfill \\ \end{array}$$	A_R–P_ is the Redlich–Peterson model isotherm constant (L g^−1^). B_R–P_ is the Redlich–Peterson model constant (mg L^−1^)^g^; $\beta$ is the Redlich–Peterson model exponent, should be 0 ≤ $\beta$ ≤ 1. Parameters were determined from the plot of ln(C_e_/Q_e_) versus ln C_e_
Jovanovic	$Q_{e} = Q_{J} \left[ {1 - exp^{{(K_{J} C_{e} )}} } \right]$	$$\begin{array}{*{20}l} {lnQ_{e} = lnQ_{max} - K_{J} C_{e} } \hfill & {\left( {21} \right)} \hfill \\ \end{array}$$	K_J_ is Jovanovic isotherm constant (L g^−1^) determined from the slope of plot of ln *q*_*e*_ against C_e_
Elovich	$\frac{{Q_{e} }}{{Q_{max} }} = K_{E} C_{e} exp\frac{{Q_{e} }}{{Q_{max} }}$	$$\begin{array}{*{20}l} {ln\left( {\frac{{Q_{e} }}{{C_{e} }}} \right) = lnK_{E } Q_{max} - \frac{{Q_{e} }}{{Q_{max} }}} \hfill & {\left( {22} \right)} \hfill \\ \end{array}$$	K_E_ is Elovich equilibrium constant (L mg^−1^) Q_max_ is Elovich maximum adsorption capacity (mg g^−1^). Elovich constants were determined from slope and intercept of the linear plot of $\ln \left( {\frac{{q_{e} }}{{C_{e} }}} \right)\;vs\;q_{e}$
Jossen	$Ce = \frac{{Q_{e} }}{H}exp\left( {Fq_{e}^{p} } \right)$	$$\begin{array}{*{20}l} {ln\left( {\frac{{C_{e} }}{{Q_{e} }}} \right) = - lnH_{J} + F_{J} Q_{e}^{p} } \hfill & {\left( {23} \right)} \hfill \\ \end{array}$$	*H* is Jossens isotherm constant (it corresponds to Henry’s constant),*p* is Jossens isotherm constant and it is characteristic of the adsorbent irrespective of temperature and the nature of adsorbents, and *F*is Jossens isotherm constant. H and F depend only on temperature Jossen’s constants were determined from the linear plot of $\ln \left( {\frac{{C_{e} }}{{q_{e} }}} \right)\;vs\;q_{e}$
Kiselev	$k_{1} C_{e} = \frac{\theta }{{\left( {1 - \theta } \right)\left( {1 + k_{n} \theta } \right)}}$	$$\begin{array}{*{20}l} {\frac{1}{{C_{e} \left( {1 - \theta } \right)}} = \frac{{K_{1} }}{\theta } + K_{1} K_{n} } \hfill & {\left( {24} \right)} \hfill \\ \end{array}$$	K_i_ is Kiselev equilibrium constant (Lmg^−1^) and K_n_ is equilibrium Constant of the formation of complex between adsorbed molecules. Kiselev constants were determined from the plot of 1/C_e_(1 − Ө) versus 1/Ө
Flory–Huggins	$\frac{{\varvec{\theta}}}{{{\varvec{C}}_{{\varvec{o}}} }} = {\varvec{K}}_{{{\varvec{FH}}}} \left( {1 - {\varvec{\theta}}} \right)^{{{\varvec{n}}_{{{\varvec{FH}}}} }}$	$$\begin{array}{*{20}l} {log\left( {\frac{\theta }{{C_{o} }}} \right) = logK_{H} + n_{H} log\left( {1 - \theta } \right)} \hfill & {\left( {25} \right)} \hfill \\ {\theta = 1 - \left( {\frac{Ce}{{C_{o} }}} \right)} \hfill & {\left( {26} \right)} \hfill \\ \end{array}$$	θ is degree of surface coverage, n_H_ is number of adsorbates occupying adsorption sites, and K_FH_ is Flory–Huggins equilibrium constant (L mol^−1^). n_FH_ and K_FH_ were determined from the linear plot of Log(θ/C_o_) versus log (1 − θ)
Fowler–Guggenheim	$K_{FG} C_{e} = \frac{\theta }{1 - \theta }exp\left( {\frac{2\theta W}{{RT}}} \right)$	$$\begin{array}{*{20}l} {ln\left[ {\frac{{Ce\;\left( {1 - \theta } \right)}}{\theta }} \right] = - lnK_{FG} + \frac{2\omega \theta }{{RT}}} \hfill & {\left( {27} \right)} \hfill \\ \end{array}$$	K_FG_ is the Fowler–Guggenheim (F–G) equilibrium constant (L mg^−1^), θ the fractional coverage, R the universal gas constant (kJ mol^−1^ K^−1^), T the temperature (K), and W is the interaction energy between adsorbed molecules (kJ mol^−1^). K_FG_ and W were determined from the linear plot of $ln \left[ {\frac{{Ce\;\left( {1 - \theta } \right)}}{\theta }} \right]$ versus $\theta$

**Table 3:** Isotherm models and their various evaluated parameters for the adsorption of Cu^2+^ onto nZVI.

Langmuir	Parameters	Freundlich	Parameters	Temkin	Parameters	DKR	Parameters	Halsey	Parameters
Q_max_ (mg g^−1^)	90.0901	k_f_	12.5401	A_T_	3.828	Q_d_	51.9873	1/n_H_	− 0.5457
K_L_ (L mg^−1^)	0.1563	1/n_f_	0.5457	B (L g^−1^)	14.678	A_DKR_	− *2* × *10*^−*7*^	n_H_	− 1.8325
R_L_	0.3591	n_f_	1.8325	b_T_ (J mol^−1^)	168.7949	E	1581.14	K_H_	0.0097
R^2^	0.9752	R^2^	0.963	R^2^	0.9586	R^2^	0.8847	R^2^	0.963

Harkin–Jura	Parameters	Redlich Peterson	Parameters	Jovanovic	Parameters	Elovich	Parameters	Jossen	Parameters
1/A_H–J_	0.0177	A_R–P_	4.69 × 10^−3^	Q_max_	13.8668	Q_max_	32.573	F	0.0307
A_HJ_	56.4972	B (L g^−1^)	2.085	K_J_	− 0.0556	K_E_	1.1007	H	1.0312
B_HJ_	1.2203	b	*5.21* × *10*^−*5*^						
R^2^	0.7025	R^2^	0.9475	R^2^	0.6105	R^2^	0.9714	R^2^	0.9714

Kiselev	Parameters	Flory–Huggins	Parameters	Fowler–Guggenheim	Parameters				
Ki	2852.4	n_H_	− 1.3651	K_FG_	2.6963				
Kn	0.6052	K_H_	0.0329	W	881.02				
*R*^*2*^	0.7566	R^2^	0.8803	R^2^	0.9487				

Langmuir isotherm model (Figure S7A) assumes no interaction of the neighboring sites, monolayer surface, identical active sites, uniformity in adsorption energy^14^. The non-linear and linear Langmuir equations are presented in Eq. (11). In this study, the Langmuir isotherm model has the highest correlation coefficient (R^2^ > 0.97) indicating the appropriateness and best fitting of equilibrium data to the Langmuir model. The Langmuir essential feature, as well as the separation factor or dimensionless constant (R_L_), was calculated using Eq. (12)^20^. Values of calculated characteristics parameters are presented in Table 3. The values of R_L_ (1 > R_L_ > 0) portrayed in Table 3 supported favorable adsorption process^35^.

Freundlich isotherm model is presented in Eq. (13) (Table 2) and the plot is as depicted in Figure S7B. The characteristic parameters are represented in Table 3. The values 12.54 and 1.83 indicated Freundlich capacity (K_F_) and intensity (n_F_) of adsorption respectively. The values of n_F_ also measure whether the adsorption is favorable or not. The value of 1/n_F_ (0.5457) less than unity and n_F_ greater than unity and less than 10 indicated a normal and favorable adsorption^36,37^. Temkin model (Eq. 14) fits the experimental data (R^2^ = 0.95) as depicted in Figure S7C. The positive value of B (14.678) and high ***b***_***T***_ (168.794 J mol^−1^) revealed the binding of Cu^2+^ onto nZVI as well as the endothermic nature of the system. A report from other researchers corroborated this^36^.

Equations (15)–(17) defined the Dubinin–Kaganer–Raduskevich (DKR) model, Polanyi potential, and adsorption free energy of DRK. DRK plot is presented in Figure S7D and Table 3 shows evaluated parameters. The DKR free energy (E = 1581.14 J mol^−1^) lower than 8 kJ mol^−1^ supported that electrostatic interaction between Cu^2+^-nZVI system is Physisorption mechanism^38^. Halsey isotherm model (Eq. 18 and plot in Figure S7E) with parameters of K_H_ and n_H_ (0.0097 and -1.8325) further supported normal and favorable adsorption indicated by the Freundlich isotherm model. However, the negative value of n_H_ couple with the low R^2^ value of Harkin-Jura (Eq. 19, Figure S7F) showed that the adsorption nature of the nZVI surface is not multilayer and heterogeneous^39^.

Combination of both the Langmuir and Freundlich isotherm attribute could be assessed in Redlich–Peterson Isotherm model (Eq. 20, Figure S7G). Redlich–Peterson correlation value (R^2^ = 0.9475) shows its versatility and fitting to equilibrium data^40,41^. Figure S7H depicts the Jovanovic isotherm (Eq. 21) model plot. Elovich isotherm model (Eq. 22, Figure S7I) has a foundation on the kinetic principle with the assumption of an increase in the adsorption sites exponentially^42^. It also takes into consideration the maximum monolayer capacity (Q_max_). Based on R^2^ value, the equilibrium data were fitted to the Elovich model but it was not as better described as compared to the Langmuir model. Also, its estimated Q_max_ = 32.573 mg g^−1^ (Table 3) being less than that of Langmuir signposted Langmuir as a better model. Jossen’s isotherm model (Eq. 23, Figure S7J) is based on a distribution of the energy of interactions between the system solid–liquid system^43^. Jossen’s fit equilibrium data with R^2^ > 0.97 (Table 3). Both Kiselev Isotherm Model (Eq. 24, Figure S7K) and the Flory–Huggins isotherm model (Eq. 25, Figure S7L) take into consideration the surface coverage (θ) of the Cu^2+^ adsorbate on the nZVI. Jovanovic isotherm model corresponds to another approximation for monolayer localized adsorption without lateral interactions which ought to be similar to the Langmuir isotherm model. However, a lower correlation coefficient (R^2^ = 0.6105) obtained in this study indicated that there is a lateral interaction and thus this model lower approach towards saturated compared to Langmuir adsorption isotherm as reported by Al-Ghouti et al.^44^. This is supported by the fit and parameters obtained from Fowler–Guggenheim (F–G) isotherm model (Eq. 27, Figure S7M). Taken into consideration is the lateral interaction of adsorption of EDC-Cu^2+^ onto nZVI by the FG- isotherm model^45^. As reported by the literature, the interaction between the adsorbed molecule is attractive, if W is positive; repulsive interaction if W is negative and no interaction between the adsorbed molecules will be observed if W = 0^42,46^. In this study, the fit of the Fowler–Guggenheim isotherm model (R^2^ = 0.9487) and positive value of W (W = 881.02 J mol^−1^) indicated that there is positive contact in the Cu^2+^-nZVI system, hence the adsorption heat increased with loading confirming endothermic adsorption process as observed in the thermodynamics studies.

The screening and arrangement are based on the understanding of the important parameters (Q_max_ and R^2^). With regards to Q_max_ (in descending order) Langmuir > DKR > Elovich > Jovanovic. With respect to R^2^ (in descending order): Langmuir > Jossen = Elovich > Freundlich = Halsey > Temkin > Fowler–Guggenheim > DKR > Flory–Huggins Kiselev > Harkin–Jura > Jovanovic. Presented in Table 4 is the comparison of maximum monolayer adsorption capacities of adsorption of Cu^2+^ onto various nano-adsorbents and nZVI used in this study. It is obvious that nZVI exceedingly surpassed other existing adsorbents reported. This indicated that nZVI is an excellent potential nano-adsorbent for effective removal of endocrine disruptive heavy metal ions.

**Table 4:** Comparison of adsorption capacity nZVIf other nano-adsorbents used for Cu^2+^ removal.

S. no.	Adsorbents	Adsorption capacity (Q_max_) (mg/g)	References
1	Pectin-iron oxide	48.99	^10^
2	Chitosan-bound Fe_3_O_4_ magnetic nanoparticles	21.5	^41^
3	Aminated polyacrylonitrile nanofiber mats	30.40	^29^
4	Carbon nanotubes	24.49	^42^
5	Carboxylmethyl-β-cyclodextrinconjugated magnetics nanoparticle	47.29	^43^
6	Fe_3_O_4_ magnetic nanoparticles coated with humic acid	46.3	^44^
7	Magnetic gamma-Fe_2_O_3_ nanoparticles coated with poly-l-cysteine	42.9	^45^
8	Magnetic nano-adsorbent modified by gum arabic	38.5	^46^
9	Hydroxyapatite nanoparticles	36.9	^47^
10	Maghemite nanoparticle	27.7	^48^
11	Amino-functionalized magnetic nanosorbent	25.77	^49^
12	Poly(hydroxyethyl methacrylate)	58.0	^50^
13	Fe_3_O_4_ nanoparticle	37.04	^51^
14	Fe_2_O_3_ nanoparticle	19.61	^51^
15	nZVI	90.09	This present study

### Adsorption kinetic with statistical error validity modeling

A kinetic study was undertaken to understand the controlling pathway, the rate of surface adsorption of the contaminant to the adsorbent, and the quantity of the adsorption capacity. The kinetics equations vis-à-vis pseudo-first-order (PFO), pseudo-second-order (PSO), Elovich, Avrami, and Power Function (Fractional power) are represented on Eqs. (28)–(34)^47^.

28 $${\text{Pseudo}}\;{\text{first-order}}\left( {{\text{Lagergren's}}\;{\text{rate}}\;{\text{equation}}} \right)\quad \ell og(q_{e} - q_{t} ) = \ell og\;q_{e} - \frac{{k_{1} t}}{2.303}$$

29 $${\text{h}}_{{1}} \;{\text{initial}}\;{\text{pseudo}}\;{\text{first-order}}\;{\text{adsorption}}\;{\text{rate}} \left( {{\text{mg}}\;{\text{g}}^{{ - 1}} \;{\text{min}}^{{ - 1}} } \right)\quad h_{1} = k_{1} q_{e}$$

30 $${\text{Pseudo}}\;{\text{second-order}}\;{\text{rate}}\;{\text{equation:}}\quad \frac{t}{{q_{t} }} = \frac{1}{{k_{2} q_{e}^{2} }} + \frac{1}{{q_{e} }}t$$

31 $${\text{h}}_{{2}} \;{\text{is}}\;{\text{the}}\;{\text{initial}}\;{\text{pseudo}}\;{\text{second-order}}\;{\text{adsorption}}\;{\text{rate:}}\quad h_{2} = k_{2} q_{e}^{2}$$

32 $${\text{Elovich}}\;{\text{model:}}\quad q_{t} = \frac{1}{\beta }\ell n\left( {\alpha \beta } \right) + \frac{1}{\beta }\ell n\left( t \right)$$

33 $${\text{Avrami}}\;{\text{model:}}\quad \ln \left( {\ln \left( {\frac{{q_{e} }}{{q_{e} - q_{t} }}} \right)} \right) = n\ln k_{Av} + n\ln t$$

34 $${\text{Power}}\;{\text{Function:}}\quad \ell og\;(q_{t} ) = \log (k) + v\log (t)$$

**Figure 2:**
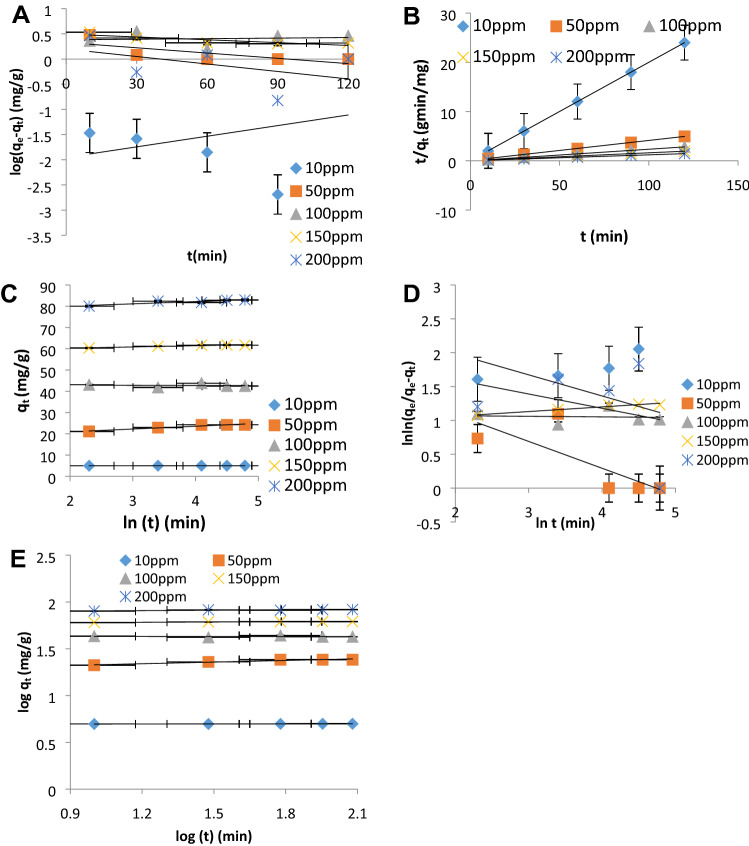
(**A**–**E**): Linearized plots of (**A**) pseudo-first-order rate equation, (**B**) pseudo-second-order rate equation, (**C**) Elovich rate equation, (**D**) Avrami kinetics models, (**E**) fractional power rate equation models for adsorption of Cu^2+^ onto nZVI at 10 ppm, 50 ppm, 100 ppm, 150 ppm and 200 ppm.

The kinetic plots are presented in Fig. 2A–E with error bars indicating the application of error models and the evaluated parameters are presented in Table 5. The kinetic constant k_1_of Pseudo first order (PFO), its adsorption rate constant h_1_, disagreement between q_e_, _exp_ and q_e_, _cal_ and low correlation coefficient, R^2^ < 0.90, demonstrated that PFO is not applicable in this study. A similar low trend in the R^2^ value was observed in the Avrami model demonstrating that it is not applicable in this study. A good agreement between the experimental quantity adsorbed (q_e_, _exp_) and the calculated quantity adsorbed (q_e_, _cal_) was observed in PSO, Elovich, and Power function. From the Elovich model, the values of ∝ (adsorption rate) increased with an increase in concentration as a result of an increase in the number of sites. The values of 1/β at 10 ppm, 50 ppm, 100 ppm, and 150 ppm are 5.882, 11.764, and 17.123 respectively. These values reflect the number of sites available for adsorption^30^. Kinetic parameters from Power Function in Table 5 indicated time-dependent of Cu(II) onto nZVI with the value of constant v less than 1 across all the concentrations. Of all these kinetic models, PSO best described the Cu(II) adsorption process and this was supported by the statistical error validity model presented in Table 5. The PSO initial adsorption rate (h_2_) increases with increase in concentration from 33.67 to 238.095 mg g^−1^ min^−1^. R^2^ values range from 0.99 to unity demonstrating the best fitting by PSO suggesting chemisorption mechanism.

**Table 5:** Kinetic data for adsorption of Cu^2+^ onto nZVI at different initial concentrations.

Kinetics model parameters	Initial Cu^2+^concentration					
	Evaluated parameters	10 ppm	50 ppm	100 ppm	150 ppm	200 ppm
**PFO**						
	q_e_,exp (mg g^−1^)	4.9859	24.1981	43.8152	61.684	81.77
	q_e_, cal (mg g^−1^)	0.01109	2.1311	2.3147	3.1261	1.5816
	k_1_ (min^−1^)	− 0.0161	8.065 × 10^−3^	− 9.212 × 10^−4^	4.375 × 10^−3^	1.152 × 10^−2^
	h_1_ (mg g^−1^ min^−1^)	1.785 × 10^−4^	1.717 × 10^−2^	2.22 × 10^−3^	1.367 × 10^−2^	1.822 × 10^−2^
	R^2^	0.1029	0.5549	0.0147	0.7275	0.2127
**PSO**						
	q_e_, exp (mg g^−1^)	4.9859	24.1981	43.8152	61.684	81.77
	q_e_, cal (mg g^−1^)	5.005	24.6305	42.5532	61.728	83.333
	k_2_ (g mg^−1^ min^−1^)	1.3441	0.02813	3.7301	3.8572	2
	h_2_ (mg g^−1^ min^−1^)	33.67	17.0648	158.73	238.095	166.667
	R^2^	1	0.9999	0.9999	1	0.9999
**Elovich**						
	qe, exp (mg g^−1^)	4.9859	24.1981	43.8152	61.684	81.77
	qe, cal (mg g^−1^)	4.9889	23.6859	42.7181	61.4962	82.3027
	α (g min^2^ mg^−1^)	6.449 × 10^+145^	1.289 × 10^+6^	− 4.39 × 10^−283^	1.3162 × 10^+44^	7.4216 × 10^+31^
	β (g min mg^−1^)	68.9655	0.755	− 15.0602	1.7274	0.9406
	R^2^	0.9397	0.9336	0.0078	0.9357	0.7967
**Avrami**						
	qe, exp (mg g^−1^)	4.9859	24.1981	43.8152	61.684	81.77
	qe, cal (mg g^−1^)	4.873	17.5472	41.307	60.1006	78.3886
	n_av_	− 0.3116	− 0.3962	− 0.0086	0.068	− 0.2113
	K_av_	2.3201	8.74 × 10^−3^	1.4047	6.977 × 10^+5^	6.9246
	R^2^	0.1457	0.5795	0.0065	0.9414	0.0852
**Fractional power**						
	qe, exp (mg g^−1^)	4.9859	24.1981	43.8152	61.684	81.77
	qe, cal (mg g^−1^)	4.9906	23.6629	42.7202	61.5025	82.2843
	v (min^−1^)	2.9 × 10^−3^	5.83 × 10^−2^	− 1.5 × 10^−3^	9.5 × 10^−3^	1.3 × 10^−2^
	k_3_ (mg g^−1^)	4.9295	18.638	42.9833	59.1561	78.0189
	k_3_v (mg g^−1^ min^−1^)	1.429 × 10^−2^	1.0866	6.447 × 10^−2^	5.619 × 10^−1^	1.0142
	R^2^	0.94	0.9311	0.0078	0.9352	0.7967

### Statistical validity of the kinetic models

Assessment on the best kinetic fitting model that is always based on linear regression coefficient could be biased inherent, hence the need for statistical validity model. The suitability, agreement, and best fit among the kinetic models are judged not only by regression coefficient (R^2^) but also with the use of statistical error validity models. Validity of kinetic data was fitted to statistical error models namely; Average relative error (ARE), Normalized Standard Deviation Δq_t_ (%), Hybrid fractional error function (HYBRD), Derivative of Marquardt's percent standard deviation (MPSD), Standard deviation of relative Error (S_RE_). The various statistical functions are presented in Table 6. Presented in Table 7 are the statistical error validity data of the kinetic models. Five statistical tools were used for the validity of these kinetic models. It is observed that the closer the agreement between the experimental quantity adsorbed (qe, *exp*) and calculated quantity adsorbed (qe, *cal*), the lower the values of these statistical tools, the better the model. In order to justify and juxtapose the best model, a reference was made to the coefficient of regression (R^2^). The higher the *R*^*2*^ values, the closer the values of qe, *exp*, and qe, *cal*, the lower the values of ∆q, *HYBRID, MPSD, ARE,* and S_RE,_ the better the kinetic models in describing the sorption process^48–50^. The values in Table 4 vividly show that pseudo-second-order at various initial Cu^2+^ concentrations (10 ppm, 50 ppm, 100 ppm, 150 ppm, and 200 ppm) best describe the sorption process. the model can be arranged in descending order with respect to *R*^*2*^: pseudo-second-order > Elovich > fractional power > Avrami > pseudo-first-order.

**Table 6:** Adsorption statistical error validity models (ASEVM)^24,43,44,46^.

Normalized standard deviation Δq_t_ (%)	$\Delta q(\% ) = 100\frac{{\sqrt {\sum\limits_{i = 1}^{n} {\left( {\frac{{q_{e,\exp } - q_{e,cal} }}{{q_{e,\exp } }}} \right)^{2} } } }}{n - 1}$	15
Hybrid fractional error function (HYBRID)	$HYBRID = \sum\limits_{i = 1}^{n} {\left[ {\frac{{\left( {q_{e,\exp } - q_{e,cal} } \right)^{2} }}{{q_{e,\exp } }}} \right]}_{i}$	16
Derivative of Marquardt’s percent standard deviation (MPSD)	${\varvec{MPSD}} = \mathop \sum \limits_{{{\varvec{i}} = 1}}^{{\varvec{n}}} \left[ {\frac{{\left( {{\varvec{q}}_{{{\varvec{e}},{\varvec{exp}}}} - {\varvec{q}}_{{{\varvec{e}},{\varvec{cal}}}} } \right)}}{{{\varvec{q}}_{{{\varvec{e}},{\varvec{exp}}}} }}} \right]^{2}$	17
Average relative error (ARE)	${\varvec{ARE}} = \mathop \sum \limits_{{{\varvec{i}} = 1}}^{{\varvec{n}}} \left[ {\frac{{{\varvec{q}}_{{{\varvec{e}},{\varvec{exp}}}} - {\varvec{q}}_{{{\varvec{e}},{\varvec{cal}}}} }}{{{\varvec{q}}_{{{\varvec{e}},{\varvec{exp}}}} }}} \right]$	21
Standard deviation of relative errors (S_RE_)	${\varvec{S}}_{{{\varvec{RE}}}} = \sqrt {\frac{{\mathop \sum \nolimits_{{{\varvec{i}} = 1}}^{{\varvec{n}}} \left[ {\left( {{\varvec{q}}_{{{\varvec{e}},{\varvec{exp}}}} - {\varvec{q}}_{{{\varvec{e}},{\varvec{cal}}}} } \right) - {\varvec{ARE}}} \right]^{2} }}{{{\varvec{n}} - 1}}}$	22

**Table 7:** Statistical Error validity data on kinetics models of adsorption of Cu(II) onto nZVI.

Adsorption statistical error validity models on kinetics of adsorption	Data at various initial Cu^2+^ concentrations				
	10 ppm	50 ppm	100 ppm	150 ppm	200 ppm
Pseudo first-order					
q_e_,exp (mg g^−1^)	4.9859	24.1981	43.8152	61.684	81.77
q_e_, cal (mg g^−1^)	0.01109	2.1311	2.3147	3.1261	1.5816
R^2^	0.1029	0.5549	0.0147	0.7275	0.2127
HYBRID	4.9637	20.1236	39.3081	55.5902	78.6374
MPSD	0.9956	0.8316	0.8971	0.9012	0.9617
ARE	0.9978	0.9119	0.9472	0.9493	0.9806
∆q	24.9444	22.7983	23.6792	23.7330	24.5165
SRE	1.98852	10.5775	20.2777	28.8043	39.6039
Pseudo second-order					
q_e_, exp (mg g^−1^)	4.9859	24.1981	43.8152	61.684	81.77
q_e_, cal (mg g^−1^)	5.005	24.6305	42.5532	61.728	83.333
R^2^	0.9986	0.9833	0.9992	0.9996	0.9989
HYBRID	7.317 × 10^−5^	0.00773	0.03635	3.1 × 10^−5^	0.02988
MPSD	1.468 × 10^−5^	0.000319	0.00083	5.09 × 10^−7^	0.000365
ARE	− 0.003830	− 0.01787	0.028803	− 0.00071	− 0.01911
∆q	0.09577	0.446729	0.72007	0.017833	0.477865
SRE	0.007634	0.2073	0.6166	0.02164	0.7719
Elovich					
q_e_,exp (mg g^−1^)	4.9859	24.1981	43.8152	61.684	81.77
q_e_, cal (mg g^−1^)	4.9889	23.6859	42.7181	61.4962	82.3027
R^2^	0.4294	0.0791	0.8596	0.9643	0.7837
HYBRID	1.8051 × 10^−6^	0.01084	0.02747	0.00057	0.00347
MPSD	3.6204 × 10^−7^	0.000448	0.000627	9.27 × 10^−6^	4.24 × 10^−5^
ARE	− 0.0006017	0.021167	0.025039	0.003045	− 0.00651
∆q	0.015042	0.529174	0.625981	0.076114	0.162865
S_RE_	0.001199	0.24556	0.53603	0.09237	0.2631
Avrami					
q_e_,exp (mg g^−1^)	4.9859	24.1981	43.8152	61.684	81.77
q_e_, cal (mg g^−1^)	4.873	17.5472	41.307	60.1006	78.3886
R^2^	0.4294	0.0791	0.8596	0.9643	0.7837
HYBRID	0.002556	1.8280	0.1436	0.04065	0.1398
MPSD	0.0005127	0.07554	0.003277	0.000659	0.00171
ARE	0.02264	0.2748	0.05724	0.02567	0.04135
∆q	0.5661	6.8713	1.4311	0.6417	1.0338
S_RE_	0.04512	3.1880	1.2255	0.7789	1.6700
Power function (fractional power)					
q_e_,exp (mg g^−1^)	4.9859	24.1981	43.8152	61.684	81.77
q_e_, cal (mg g^−1^)	4.9906	23.6629	42.7202	61.5025	82.2843
R^2^	0.5443	0.5827	0.7156	0.6609	0.6989
HYBRID	4.43 × 10^−6^	0.01184	0.02737	0.00053	0.00323
MPSD	8.886 × 10^7^	0.000489	0.000625	8.66 × 10^−6^	3.96 × 10^−5^
ARE	− 0.000943	0.022117	0.024991	0.002942	− 0.00629
∆q	0.0236	0.5529	0.6247	0.07356	0.15724
S_RE_	0.001878	0.2565	0.535004	0.089279	0.254005

### Adsorption mechanisms for sorption of Cu^2+^ onto nanoscaled zerovalent iron (nZVI)

**Figure 3:**
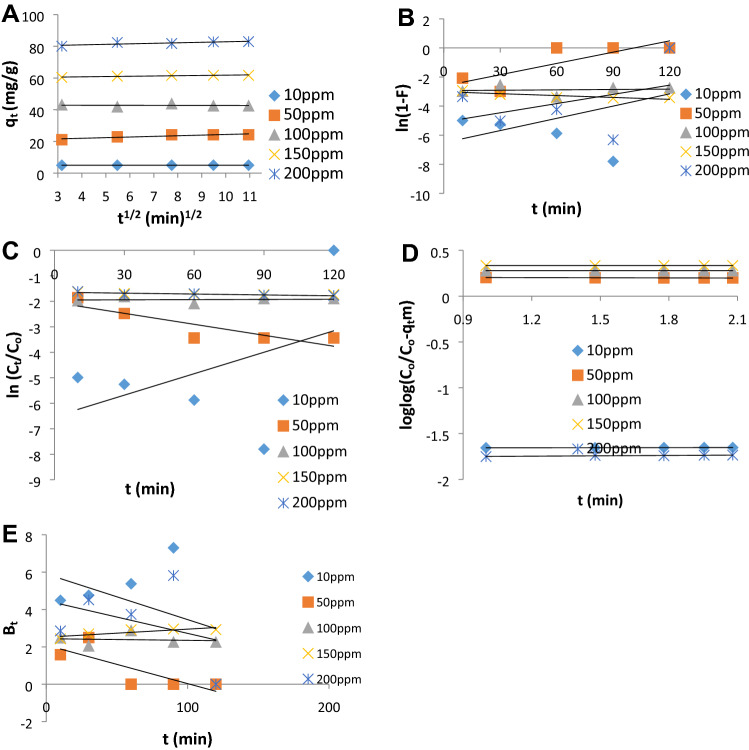
Linearized plots of (**A**) intraparticle diffusion, (**B**) liquid film diffusivity, (**C**) external diffusion, (**D**) Bangham and (**E**) Boyd models for adsorption of Cu^2+^ onto nZVI at 10 ppm, 50 ppm, 100 ppm, 150 ppm and 200 ppm.

Figure 3A–E show the linear plots of intraparticle diffusion, liquid diffusion, external diffusion, Bangham and Boyd models. Adequate understanding of the adsorption mechanism is enhanced by the determination of the rate-controlling/determining step. The three definite steps that could be used to describe the adsorption rate are^51^: (1) Intraparticle or pore diffusion, where adsorbate molecules percolate into the interior of adsorbent particles, (2) Liquid film or surface diffusion where the adsorbate is transported from the bulk solution to the external surface of the adsorbent, and (3) adsorption on the interior sites of the sorbent. Since the plot of Intraparticle diffusion (Fig. 3A) did not pass through the origin, it is demonstrated that it is not the only rate-determining step^52^. Other mechanisms such as surface diffusion and external diffusion also participated in the mechanism of Cu(II) removal. However, the higher R^2^ values of intraparticle diffusion from the evaluated parameters presented in Table 8 demonstrated that the mechanism is pore diffusion dependent which was confirmed by Bangham and scattered plot of Boyd models^53,54^. The intercept of intraparticle diffusion which is the thickness of the surface gives information about the contribution of the surface sorption in the rate-determining step. The larger the intercept, the greater the contribution of nZVI in adsorption of Cu^2+^ as observed from the trend across the concentrations investigated.

**Table 8:** Adsorption Mechanism models for Sorption of Cu^2+^ onto nZVI.

Adsorption mechanism models	Cu^2+^ concentration				
	10 ppm	50 ppm	100 ppm	150 ppm	200 ppm
Intraparticle diffusion					
k_ip_ (mg g^−1^ min^−0.5^)	0.0047	0.3993	− 0.0199	0.1743	0.3224
C	4.9501	20.378	42.883	60.052	79.634
R^2^	0.9795	0.8321	0.0069	0.8322	0.7185
Liquid film diffusion					
K_LFD_	− 0.0281	− 0.0259	− 0.0009	0.0043	− 0.0211
C	− 6.5233	− 2.6199	− 2.9337	− 3.0157	− 5.0927
R^2^	0.1855	0.6484	0.0147	0.7275	1.552
External diffusion					
k_ext_	− 0.0281	0.0143	0.0003	0.0008	0.0012
C	− 6.5233	− 2.0416	− 0.0119	− 1.6533	− 1.6452
R^2^	0.1855	0.7597	0.0119	0.7058	0.6417
Bangham					
ἅ	0.003	− 0.0047	0.0004	0.00008	0.0133
Ko	0.025285	1.8564	2.1820	2.4858	0.019896
R^2^	0.9404	0.9344	0.0078	0.9351	0.7968
Boyd					
R^2^	0.1623	0.6170	0.0147	0.7275	0.1258

### Thermodynamics analysis

Thermodynamics analysis is imperative to determine the (enthalpy change) heat content (ΔH); entropy change (degree of randomness, ΔS), possibility, and spontaneity (Gibbs free energy change, ΔG) in every adsorption process. The plots in thermodynamics studies are presented in the supplementary document associated with this article. As observed in Figure S8, intensification in the percentage removal efficiency was attained with an increase in temperature of the system supporting the endothermic process. This is due to a decrease in the mass transfer resistance and boundary layer thickness of nZVI^55^. Van’t Hoff’s linear plot of log Kc against 1/T was portrayed in Figure S9 and the result obtained was presented in Table 9. The positive value of ΔH (+ 50.6059 kJ mol^−1^) confirmed that the adsorption process is endothermic in nature^56^. The positive value of ΔS (+ 174.679 J mol^−1^ K^−1^) shows an increase in the degree of randomness of the lateral interaction during the adsorption of Cu^2+^ at the solid/liquid interface. This could be enhanced by the appropriate stirring speed. The feasibility and spontaneity of the adsorption process are confirmed by the negative values of ΔG (− 1.6765 to 7.9602 kJ mol^−1^).

**Table 9:** Thermodynamic parameters for adsorption of Cu^2+^ onto nZVI.

T(K)	ΔG (kJ mol^−1^)	ΔH (kJ mol^−1^)	ΔS(J mol^−1^ K^−1^)	Kc
298	− 1.6765	+ 50.6059	174.679	1.96711
308	− 2.8163			3.00304
318	− 4.4575			5.39623
328	− 7.7182			16.9419
338	− 7.9602			16.9825

### Desorption mechanism

**Figure 4:**
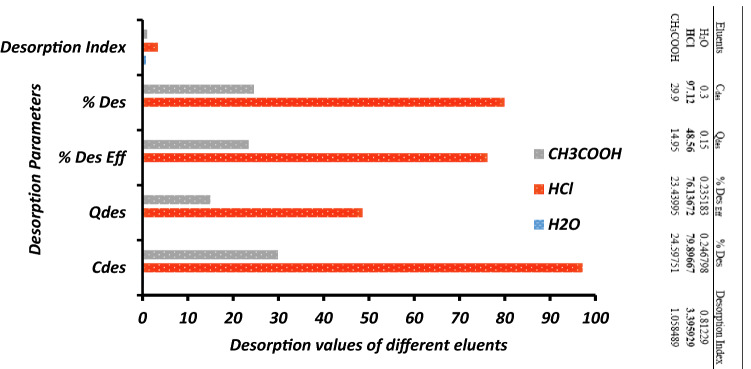
Comparative effect of different eluents in the desorption of Cu^2+^ from Cu^2+^-loaded nZVI.

Figure 4 shows the comparative effect of different eluents in the desorption of Cu^2+^ from Cu^2+^-loaded nZVI. The opportunity to investigate regeneration and reusability of loaded adsorbent is enhanced by desorption studies. The effectiveness of three different eluents and desorbing agents (HCl, CH_3_COOH, and H_2_O) was investigated. The basic desorption mechanisms are ion exchange, complexation, and precipitation depending on the most effective desorbing agent^57^. The exact mechanism involved in the adsorption process is revealed by the performance of the most effective desorbing agent. The maximum percentage of Cu(II) desorbed from Cu(II) loaded-nZVI using HCl was 79.89% showing the best desorption index of 3.39. The effectiveness of HCl as the best desorbing agent among the three eluents used is supported by the findings of Reddiar et al. (2019)^50^ Acetic acid also performed averagely while distilled-deionized water was a poor desorbing agent in the desorption of Cu(II) from Cu(II)-loaded-nZVI. Thus, the adsorption of Cu(II) onto nZVI is routed by ion exchange. Ion-exchange, electrostatic and physiochemical mechanistic nature of the adsorption supported by the previous studies^50,58,59^.

## Conclusion

This study revealed the effectiveness of nZVI as an auspicious nano sorbent for the efficient elimination of endocrine disruptive heavy metal ions. The quality physicochemical properties of nZVI gave it an edge among the list of other nano-adsorbents compared. Evidence of the adsorption of Cu^2+^ onto nZVI was revealed by a change in morphology and elemental distribution by SEM and EDX respectively from post adsorption characterization. The adsorption of Cu^2+^ onto nZVI was well influenced by operational parameters. Optimum adsorption was achieved at pH 6 with 98.31% removal efficiency, 73.73 mg g^−1^ quantity adsorbed and 200 rpm stirring speed. Thermodynamics parameters ΔH° (+ 50.6059 kJ mol^−1^), ΔS° (174.6790 J mol^−1^ K^−1^), ΔG° (− 1.6765 kJ mol^−1^ to − 7.9602 kJ mol^−1^). Indicated random, feasible, spontaneous, and endothermic nature of the adsorption process. The adsorption behavior was well explained by the Langmuir isotherm model and it followed the following order: Langmuir > Jossen/Elovich > Freundlich/Halsey > Temkin > Fowler–Guggenheim > Redlich–Peterson > DKR > Flory–Huggins > Kiselev > Harkin–Jura > Jovanovic. Langmuir best described equilibrium data. The Langmuir monolayer adsorption capacity (90.09 mg g^−1^) surpassed other nano-adsorbents utilized for the adsorption of Cu(II) ion. The Pseudo-second-order (PSO) best described the kinetics model based on R^2^ values greater than 0.99, close agreement between qe, exp and qe, cal and lower values of the five rigorous statistical validity models (Δq_t,_ ARE, HYBRD, MPSD, and S_RE_). The mechanism model was pore diffusion dependent. Best desorption capacity and the index was portrayed by HCl indicating that ion-exchange, electrostatic, and physisorption mechanism. Based on the capacity displayed by nZVI in adsorption of EDC Cu^2+^, it could be recommended for effective industrial treatment of heavy metal ions.

## Supplementary Information

Supplementary Information.

## Data Availability

Available data are presented in the study and no other data were used to support the study.
