# Supplementary material for: Two–three parameters isotherm modeling, kinetics with statistical validity, desorption and thermodynamic studies of adsorption of Cu(II) ions onto zerovalent iron nanoparticles
Source: Sci Rep. 2021 Aug 12;11:16454. doi: 10.1038/s41598-021-95090-8 (PMC8361154; doi:10.1038/s41598-021-95090-8)
Supplement: Supplementary file 1 — Supplementary Information. [file 41598_2021_95090_MOESM1_ESM.docx]

**Two-Three Parameters Isotherm Modeling, Kinetics with Statistical Validity, Desorption and Thermodynamic Studies of Adsorption of Cu(II) ions onto Zerovalent Iron Nanoparticles**

**Adewumi O. Dada^1,6,7*^, Folahan A. Adekola^2^, Ezekiel O. Odebunmi^3^, Adeniyi S. Ogunlaja^4^, Olugbenga S. Bello^1,5^**

^1*^Industrial Chemistry Programme, Nanotechnology Laboratory, Department of Physical Sciences, Landmark University, P.M.B.1001, Omu-Aran, Kwara State, Nigeria.

^2^Department of Industrial Chemistry, University of Ilorin, P.M.B. 1515, Nigeria.

^3^Department of Chemistry, University of Ilorin, P.M.B. 1515, Nigeria.

^4^Department of Chemistry, Nelson Mandela Metropolitan University, P.O. Box 77000, Port Elizabeth 6031, South Africa

^5^Department of Pure and Applied Chemistry, Ladoke Akintola University of Technology, P.M.B 4000, Ogbomoso, Oyo State, Nigeria

^6^Sustainable Development Goal (SDG) group 6: Clean Water and Sanitation

^7^Sustainable Development Goal (SDG) group 11: Sustainable Cities and Communities

*Corresponding author: [dada.oluwasogo@lmu.edu.ng](mailto:dada.oluwasogo@lmu.edu.ng)

**Supplementary Material**

**Detailed Procedures**

**S2.1. Synthesis of Zerovalent Iron nanoparticles (nZVI)**

The synthesis of zerovalent iron nanoparticles for the removal of endocrine disruptive heavy metal ion was carried out using bottom-up approach in a single pot system. the procedure described in our previous studies (Dada et al., 2015; Dada et al., 2017;Dada et al., 2016). Under anaerobic environment, a resulting black colouration of core shell zerovalent iron nanoparticles (nZVI) was obtained from the reaction between 0.023 M solution of FeCl_3_.6H_2_O and 0.125 M solution of NaBH_4_ in ratio 1:5. Described in Eq. 1 is the reaction of the single-pot synthesis by bottom -up approach via chemical reduction:

4Fe^3+^ + 3BH_4_^−^ + 9H_2_O → 4Fe^0^↓+3H_2_BO_3_^−^ + 12H^+^ + 6H_2_↑ (1).

**S2.2. Surface Charge (pH_pzc_), Surface Area, Pore Width and Volume Determination**

The surface charge is the point of zero charge (PZC). This was determined following the procedure in our previous studies and other report in the literature (Postai et al., 2016; Dada et al., 2017; Mtshatsheni et al., 2019). It was carried out by pH variation from 2 to 12. pH adjustments were achieved with 0.1 M HNO_3_ or 0.1 M NaOH. The analysis on the determination of surface area, pore size, width and volume were performed using Micrometritics AutoChem II Chemisorption Analyzer by Brunauer-Emmett-Teller (BET) and Barrett-Joyner-Halenda (BJH) methods

**S2.3. Effect of Stirring Speed, pH and Co-existing Ions**

In order to optimize the stirring speed (agitation speed), five different speeds were chosen from 160 – 240 rpm at optimum conditions. The pH of each of the solutions was varied to the desired value with 0.1 M NaOH and 0.1 M HNO_3_ solutions. The investigation of the effect of co-existing ions/ionic strength (salinity) on Cu^2+^ adsorbed onto nZVI was carried out using NaCl solution of various concentrations from 0.001 M to 1.0 M (Dada et al., 2017; Liu et al., 2018; Ramirez et al., 2020)

**S2.4. Surface morphology and Elemental Distribution**

The surface morphological characterization and elemental analysis were carried out using a Scanning Electron Microscopy (SEM) integrated with Energy Dispersive X-ray (EDX) analyzer. SEM images and EDX spectra were obtained using a TESCAN Vega TS 5136LM typically at 20 kV at a working distance of 20 mm. Samples for SEM analysis were prepared by coating them in gold using a Balzers’ Spluttering device.

Supplementary Figures Relevant to Results and Discussion

Figure S1. Determination of isoelectric point (Points of zero-charge) of nZVI

Figure S2: Effect of initial Concentration on adsorption of Cu(II) ion onto nZVI

Figure S3: Effect of contact time on Cu^2+^ adsorbed onto nZVI.

.

Figure S4: The effect of pH on adsorption of Cu^2+^ onto nZVI

Figure S5: Effect of Ionic strength on adsorption of Cu^2+^onto Nzvi

Figure S6.Effect of Stirring speed on adsorption of Cu^2+^ onto nZVI

List of Supplementary Figures for Isotherm models

­

Supplementary Figures (S7A – S7M): Linear plots of Langmuir (S7A) Freundlich (S7B) Temkin (S7C) DKR (S7D) Halsey (S7E) Harkin-Jura (S7F) Redlich Peterson, (S7G) Jovanovic (S7H) Elovich, (S7I) Jossen,(S7J) Kiselev (S7K) Flory-Huggins (S7L) and Fowler-Guggenheim (S7M) isotherm Models of Adsorption of Cu(II) onto nZVI

Plots from Thermodynamics Studies

Figure S8: Effect of Temperature on Adsorption of Cu^2+^ onto nZVI. Figure S9: Van’t Hoff plot for the adsorption of Cu^2+^ onto nZVI
